# Changes in the investigation and management of suspected myocardial infarction and injury during COVID-19: a multi-centre study using routinely collected healthcare data

**DOI:** 10.3389/fcvm.2024.1406608

**Published:** 2024-05-21

**Authors:** Lara Chammas, Kevin Yuan, Stephanie Little, Gail Roadknight, Kinga A. Varnai, Shing Chan Chang, Shirley Sze, Jim Davies, Andrew Tsui, Hizni Salih, Ben Glampson, Dimitri Papadimitriou, Abdulrahim Mulla, Kerrie Woods, Kevin O’Gallagher, Anoop D. Shah, Bryan Williams, Folkert W. Asselbergs, Erik Mayer, Richard Lee, Christopher Herbert, Tom Johnson, Stuart Grant, Nick Curzen, Ajay M. Shah, Divaka Perera, Riyaz S. Patel, Keith M. Channon, Amit Kaura, Jamil Mayet, David W. Eyre, Iain Squire, Raj Kharbanda, Andrew Lewis, Rohan S. Wijesurendra

**Affiliations:** ^1^ Nuffield Department of Population Health, University of Oxford, Oxford, United Kingdom; ^2^ Oxford University Hospitals NHS Foundation Trust, John Radcliffe Hospital, Oxford, United Kingdom; ^3^ NIHR Imperial Biomedical Research Centre, Imperial College London and Imperial College Healthcare NHS Trust, London, United Kingdom; ^4^ NIHR Oxford Biomedical Research Centre, Oxford University Hospital NHS Foundation Trust, John Radcliffe Hospital, Oxford, United Kingdom; ^5^ NIHR Biomedical Cardiovascular Research Centre, Glenfield Hospital, Leicester and the University of Leicester, Leicester, United Kingdom; ^6^ Department of Computer Science, University of Oxford, Oxford, United Kingdom; ^7^ NIHR King’s Biomedical Research Centre, King’s College London and King’s College Hospital NHS Foundation Trust, London, United Kingdom; ^8^ NIHR University College London Biomedical Research Centre, University College London and University College London Hospitals NHS Foundation Trust, London, United Kingdom; ^9^ Institute of Health Informatics, University College London, London, United Kingdom; ^10^ Department of Cardiology, Amsterdam Cardiovascular Sciences, Amsterdam University Medical Centre, University of Amsterdam, Amsterdam, Netherlands; ^11^ NIHR BRC at the Royal Marsden and Institute of Cancer Research, London, United Kingdom; ^12^ NIHR Leeds Clinical Research Facility, Leeds Teaching Hospitals Trust and University of Leeds, Leeds, United Kingdom; ^13^ NIHR Bristol Biomedical Research Centre, University of Bristol and University Hospitals Bristol and Weston NHS Foundation Trust, Bristol, United Kingdom; ^14^ NIHR Manchester Biomedical Research Centre, Manchester University NHS Foundation Trust and the University of Manchester, Manchester, United Kingdom; ^15^ NIHR Southampton Clinical Research Facility and Biomedical Research Centre, Faculty of Medicine, University of Southampton and University Hospital Southampton NHS Foundation Trust, Southampton, United Kingdom; ^16^ NIHR Guys & St Thomas’ Hospital Clinical Research Facility, King’s College Hospital, and King’s College London British Heart Foundation Centre of Excellence, London, United Kingdom; ^17^ Division of Cardiovascular Medicine, Radcliffe Department of Medicine, University of Oxford, Oxford, United Kingdom

**Keywords:** troponin, COVID-19, emergency department, myocardial infarction, myocardial injury

## Abstract

**Objective:**

The COVID-19 pandemic was associated with a reduction in the incidence of myocardial infarction (MI) diagnosis, in part because patients were less likely to present to hospital. Whether changes in clinical decision making with respect to the investigation and management of patients with suspected MI also contributed to this phenomenon is unknown.

**Methods:**

Multicentre retrospective cohort study in three UK centres contributing data to the National Institute for Health Research Health Informatics Collaborative. Patients presenting to the Emergency Department (ED) of these centres between 1st January 2020 and 1st September 2020 were included. Three time epochs within this period were defined based on the course of the first wave of the COVID-19 pandemic: pre-pandemic (epoch 1), lockdown (epoch 2), post-lockdown (epoch 3).

**Results:**

During the study period, 10,670 unique patients attended the ED with chest pain or dyspnoea, of whom 6,928 were admitted. Despite fewer total ED attendances in epoch 2, patient presentations with dyspnoea were increased (*p* < 0.001), with greater likelihood of troponin testing in both chest pain (*p* = 0.001) and dyspnoea (*p* < 0.001). There was a dramatic reduction in elective and emergency cardiac procedures (both *p* < 0.001), and greater overall mortality of patients (*p* < 0.001), compared to the pre-pandemic period. Positive COVID-19 and/or troponin test results were associated with increased mortality (*p* < 0.001), though the temporal risk profile differed.

**Conclusions:**

The first wave of the COVID-19 pandemic was associated with significant changes not just in presentation, but also the investigation, management, and outcomes of patients presenting with suspected myocardial injury or MI.

## Introduction

The COVID-19 pandemic resulted in substantial excess mortality and disruption to usual patterns of healthcare utilisation worldwide. Early in the pandemic, there was a reduction in the number of patients presenting with acute coronary syndromes (ACS) and myocardial infarction (MI) in the UK (1, 2), Europe (3–5) and the USA (6, 7). There was also higher mortality and/or major adverse cardiac events following non-ST-segment-elevation MI during the initial spread and first peak of the pandemic in the USA (8) and elsewhere (9), and a decrease in the rate of acute cardiovascular admissions during the first weeks of a COVID-19 lockdown (10).

In the UK, the first COVID-19 fatality was reported on 5th March 2020, with physical distancing measures encouraged on 16th March 2020. A nationwide lockdown was introduced on 26th March 2020. There was a reduction in the weekly number of patients with ACS admitted to hospital in England by the end of March 2020 (1), which was partly reversed by the end of May 2020 (11). This observation may have reflected patient factors, including delayed or deferred presentations to healthcare providers, perhaps reflecting concern regarding the risk of nosocomial COVID-19 infection and/or as a response to public health messaging.

Significant changes were also introduced to protocols for the triage, diagnosis, and management of patients with suspected and confirmed MI (12–16), driven by urgent and simultaneous changes in bed capacity, staffing availability amidst redeployment, new infection control protocols, and other factors (5). Furthermore, medical literature at the time suggested a high risk of and from cardiovascular involvement from COVID-19 in hospitalised patients (17–20), which might have driven altered clinician behaviour.

Less information is available on whether there were also changes in clinician behaviour related to the investigation and subsequent management of patients with suspected MI. We hypothesised that the first lockdown period of the COVID-19 pandemic in the UK, from March to June 2020, was associated with changes in the presentation, investigation, and management of suspected MI or myocardial injury. In order to address this hypothesis, we utilised routinely collected data from three major cardiac centres during the first eight months of 2020, comprising over 79,000 Emergency Department (ED) attendances from over 50,000 unique patients. We focused on patients presenting with chest pain and with dyspnoea; we included the latter group both because patients with MI can present primarily with dyspnoea, and to capture COVID-related practice, including any changes in the use of troponin testing. Unlike national registry data, routinely collected healthcare data from electronic medical records incorporate granular individual patient data, allowing detailed insight into factors underlying the changes in presentation and management of patients.

## Materials and methods

### Data sources and patient selection

The National Institute for Health Research (NIHR) Health Informatics Collaborative (HIC) project facilitates re-use of anonymised, routinely captured clinical data for translational research (21). This project was approved by the HIC Cardiovascular/COVID-19 Theme Scientific Steering Group Meeting in March 2021. The overall study received a favourable ethical opinion from London-South East Research Ethics Committee (REC reference 16/HRA/3327).

We obtained routinely collected healthcare data from three tertiary centres with EDs that collaborate in the HIC and had the necessary data available (Imperial College Healthcare, University College Hospital, and Oxford University Hospitals NHS Foundation Trusts). The study time period was 1st January 2020 to 1st September 2020; these data were released as part of a COVID-19 dataset. Data were extracted on demographics, presenting complaint, troponin measurement, COVID status, inpatient admission, and subsequent cardiac procedures (including diagnostic cardiac catheterisation, percutaneous coronary intervention, and coronary artery bypass graft surgery). Data on survival were obtained from linkage to the NHS spine prior to de-identification of all data in the trusted research environment (21).

### Time epochs

ED attendances and admission data were available on an aggregated weekly basis, commencing from Wednesday 1st January 2020. The study period was divided into three time epochs based on these weekly data as follows: 1st January 2020–10th March 2020 inclusive (epoch 1); 11th March 2020–16th June 2020 (epoch 2); and 17th June 2020–1st September 2020 (epoch 3). The epochs were defined based on the time course of the first wave of the COVID-19 pandemic in the UK and associated national restrictions. The UK Prime Minister announced on 16th of March 2020 that all citizens should “stop non-essential contact and travel”; together with prior widespread media coverage, this may have resulted in behavioural changes prior to the official national lockdown on 23rd March 2020. We therefore commenced epoch 2 on 11th March 2020 (the epoch had to begin on a Wednesday due to the aggregated weekly study data). Epoch 3 commenced on 17th June 2020 as this was when the national lockdown ended, with reopening of non-essential retail outlets.

### Complaint codes

SNOMED CT codes were used to identify participants attending the ED with a primary complaint of “chest pain” (29857009), “difficulty breathing” (230145002), and “dyspnoea” (267036007); the latter two categories are amalgamated as “dyspnoea” in the remainder of this manuscript.

### Troponin measurements

Each centre measured troponin I or troponin T using either contemporary or high sensitivity assays, as previously described (22). Analyses were undertaken to identify all troponin tests done within one day of the relevant ED attendance. Participants with ≥1 test >40 ng/L were classified as “troponin positive”, whereas those in whom all test results were ≤40 ng/L were classified as “troponin negative”. This cut-off was chosen pragmatically, as it was not possible to adjust for the small differences in reference ranges for the different commercial troponin assays used (22).

### Cardiac procedures

The operating procedure codes supplement (OPCS) was used to define the following categories of cardiac procedures: diagnostic cardiac catheterisation (K631, K632, K633, K634, K635, K636), percutaneous coronary intervention (K491, K492, K493, K502, K504, K508, K512, K518, K651, K751, K752, K753, K754), and coronary artery bypass surgery (K401, K402, K403, K404, K408, K411, K451, K452, K453, K454, K471, K478, K479).

### Statistical analysis

Patient descriptive statistics are displayed as medians with interquartile range (IQR) for age, and as absolute numbers for categorical variables.

The number of attendances to the ED each week is the absolute number, allowing for repeat presentation by an individual patient. Troponin tests were matched to ED attendance by patient unique identifier and hospital name, filtering for tests conducted more than one day before the attendance date or one day after the departure date from the ED. The number of troponin tests was the absolute number of tests conducted in the ED that week. The troponin testing rate is the number of unique patients receiving a troponin test weekly. The number of cardiac procedures was calculated by counting the number of procedures of interest conducted at the hospitals each week.

Figures showing weekly means were plotted using the “geom_smooth” function of the R package “ggplot”, with the method loess and a span of 0.3 to show the smoothed conditional weekly means. Statistical difference between the weekly values and rates between epoch one and epoch two were calculated using a Wilcox signed-rank test.

Kaplan–Meier survival curves were calculated and plotted using the R packages “survival” and “jskm”. Survival time was calculated as the number of days between attendance to the ED and date of death (from any cause) or censorship, restricted to a maximum of either 30 or 250 days (with a landmark analysis at 30 days in the latter groups). A log-rank test was used to calculate the statistical difference in survival rates between groups. The proportional hazard test (23) as implemented by the cox.zph command was used to test the proportional hazards assumption against non-proportional hazards (that is, time-varying coefficients). Two-sided *P*-values <0.05 were considered significant.

### Patient and public involvement

This study involved the secondary use of existing data sources and did not include patients as study participants. No patients were involved in setting the research question, the study design, or the overall conduct of this study.

## Results

### Study population

**Figure 1:**
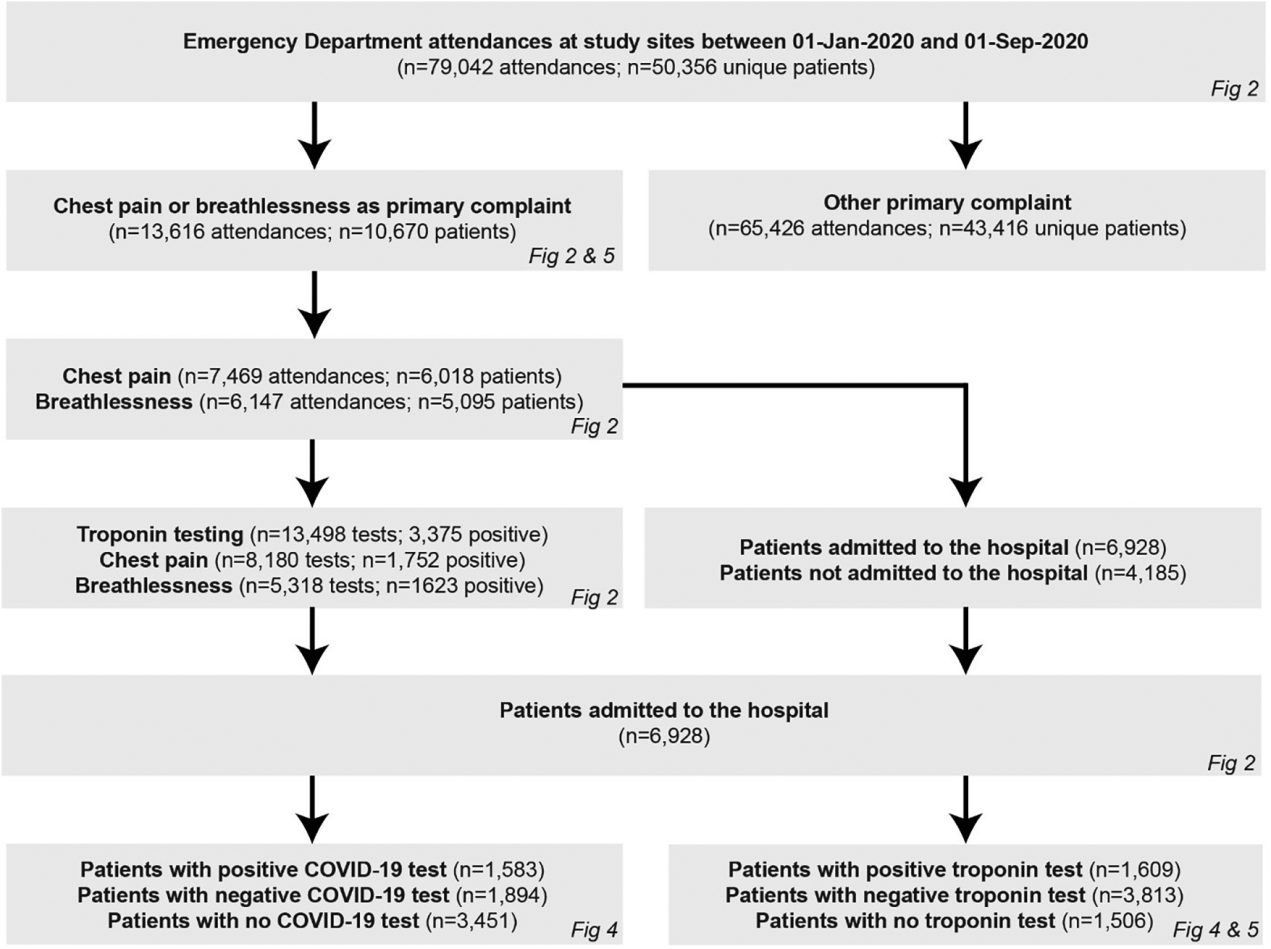
Patient cohort diagram. Consort diagram indicating numbers of patients in the study.

A patient cohort diagram is shown in Figure 1.

A total of 50,356 patients had a combined total of 79,042 ED attendances across the study sites during the study period (30,281 attendances for 18,587 patients at Imperial College Healthcare, 19,091 attendances for 11,753 patients at University College Hospital, and 29,670 attendances for 20,016 patients at Oxford University Hospitals).

Of these, 13,616 attendances in 10,670 unique patients were for chest pain (7,469 attendances in 6,018 unique patients) or dyspnoea (6,147 attendances in 5,095 unique patients) as the primary complaint, with an average of 1.1 and 0.9 troponin tests per attendance with chest pain and dyspnoea, respectively. A greater proportion of troponin tests were positive in patients with dyspnoea (5,318 tests of which 1,623 positive, 30.5%) compared to chest pain (8,180 tests of which 1,752 positive, 21.4%, *p* < 0.001). Overall, 6,928 hospital admissions resulted from these 13,616 attendances (51% admission rate), with 1,609 admissions (23%) for patients with a positive troponin and 1,538 admissions (23%) for patients with a positive COVID-19 test. The baseline characteristics of the 10,670 unique patients who attended the ED with chest pain and dyspnoea during the study period are shown in Table 1 and the corresponding characteristics for the 6,928 patients who went on to be admitted are shown in Table 2; in both cases, these baseline characteristics were largely consistent between the time epochs with no significant differences in sex, ethnicity, or social deprivation index across the time cohorts.

**Table 1:** Baseline characteristics of patients presenting to emergency department with chest pain and dyspnoea, split by time epoch.

Epoch	Unique patients	Median age, years (IQR)	Sex (*n*, %)	Ethnicity (*n*, %)	IMD quintile (*n*, %)
1	3,655 (366 per week)	66 (28)	Female (1,770, 48%)	White (2,278, 62%)	1 (719, 20%)
			Male (1,885, 52%)	Asian (270, 7%)	2 (901, 25%)
				Black (280, 8%)	3 (648, 18%)
				Other (426, 12%)	4 (648, 18%)
				Not known (401, 11%)	5 (580, 16%)
					Unknown (150, 4%)
2	4,294 (307 per week)	65 (28)	Female (2,013, 47%)	White (2,547, 60%)	1 (836, 20%)
			Male (2,281, 53%)	Asian (279, 7%)	2 (1,005, 23%)
				Black (359 8%)	3 (808, 19%)
				Other (541, 13%)	4 (769, 18%)
				Not known (568, 13%)	5 (703, 16%)
					Unknown (173, 4%)
3	2,721 (247 per week)	65 (28)	Female (1,290, 47%)	White (1,712, 63%)	1 (500, 18%)
			Male (1,431, 53%)	Asian (182, 7%)	2 (672, 25%)
				Black (213, 8%)	3 (465, 17%)
				Other (303, 11%)	4 (496, 18.2%)
				Not known (311, 11%)	5 (494, 18%)
					Unknown (94, 3%)

**Table 2:** Baseline characteristics of patients admitted after presenting to emergency department with chest pain and dyspnoea, split by time epoch.

Epoch	Unique patients	Median age, years (IQR)	Sex (*n*, %)	Ethnicity (*n*, %)	IMD quintile (n, %)
1	2,356 (236 per week)	70 (27)	Female (1,138, 48%)	White (1,482, 63%)	1 (446, 19%)
			Male (1,218, 52%)	Asian (155, 7%)	2 (610, 26%)
				Black (170, 7%)	3 (418, 18%)
				Other (290, 12%)	4 (408, 17%)
				Not known (259, 11%)	5 (384, 16%)
					Unknown (90, 4%)
2	2,896 (207 per week)	69 (26)	Female (1,248 43%)	White (1,651, 57%)	1 (569, 20%)
			Male (1,648, 57%)	Asian (184, 6%)	2 (706, 24%)
				Black (259, 9%)	3 (540, 19%)
				Other (427, 13%)	4 (523, 18%)
				Not known (410, 14%)	5 (459, 16%)
					Unknown (99, 3%)
3	1,676 (152 per week)	70 (25)	Female (759, 45%)	White (1,065, 64%)	1 (309 18%)
			Male (917, 55%)	Asian (113, 7%)	2 (409, 24%)
				Black (117, 7%)	3 (307, 18%)
				Other (185, 11%)	4 (306, 18%)
				Not known (196, 12%)	5 (292, 17%)
					Unknown (53, 3%)

### Temporal changes in ED attendance and troponin testing during COVID-19

**Figure 2:**
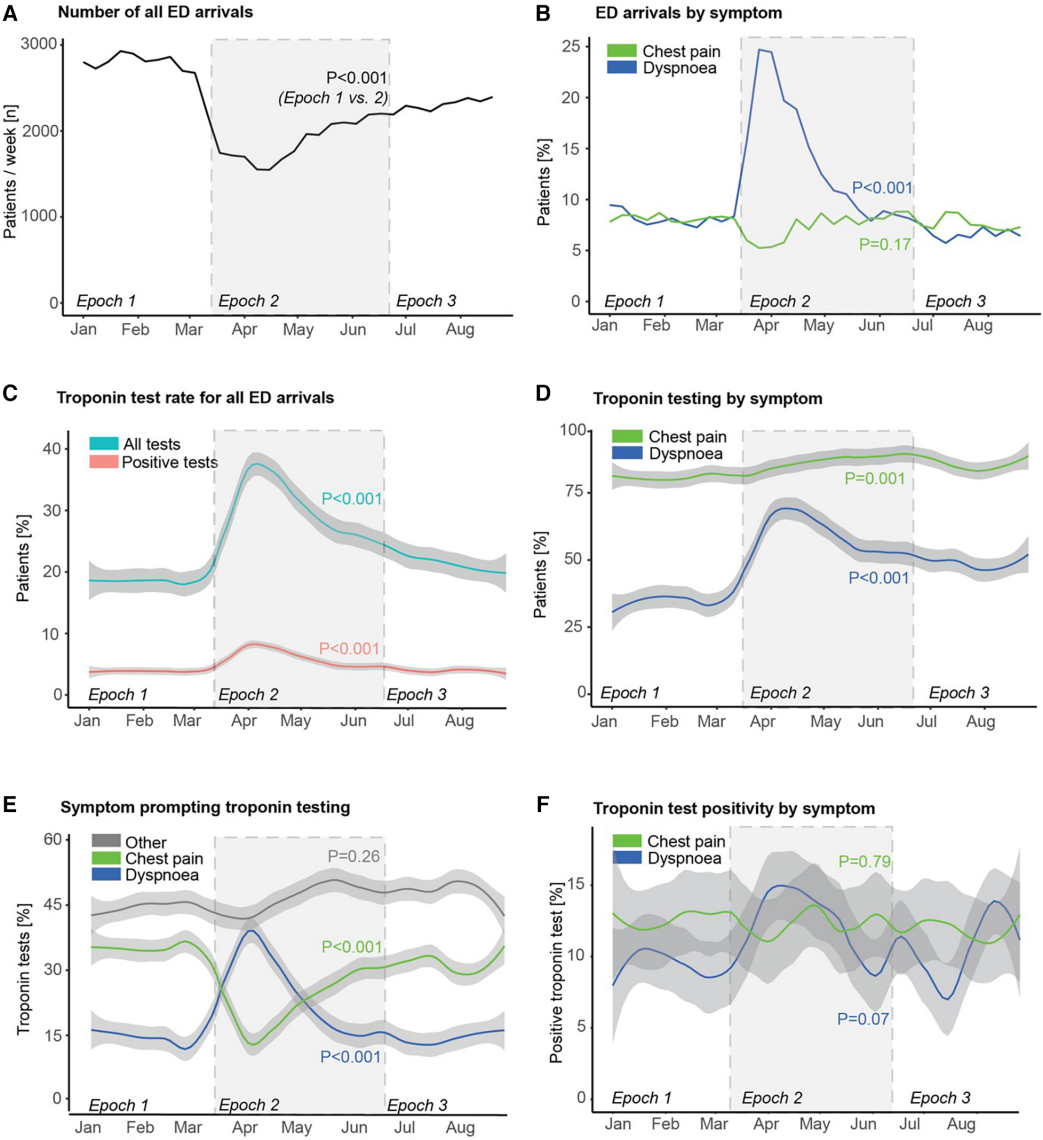
Emergency department attendance, troponin testing, and troponin positivity rates during the first wave of COVID-19. Trends of (**A**) number of all emergency department (ED) arrivals, (**B**) proportion of ED arrivals stratified by primary presenting symptom/complaint, (**C**) proportion of ED arrivals undergoing troponin testing, and proportion with a positive troponin test, (**D**) proportion of patients attending ED with either chest pain or dyspnoea undergoing troponin testing, (**E**) primary symptom in patients undergoing troponin testing, and (**F**) rate of troponin test positivity stratified by primary presenting symptom/complaint. *P*-values reflect comparison between epoch 1 and epoch 2 in each case.

There was a significant reduction in the rate of ED attendance for any complaint in epoch 2 compared to epoch 1 (*p* < 0.001; Figure 2A), with some recovery in epoch 3 (*p* < 0.001 vs. epoch 2), but still remaining below epoch 1 (*p* < 0.001). Whilst the proportion of all ED attendances due to chest pain was similar from epoch 1 to epoch 2 (*p* = 0.17), the proportion due to dyspnoea rose rapidly from epoch 1 to epoch 2 (*p* < 0.001; Figure 2B), peaking at almost 25% of all attendances before falling back to the baseline level of <10% by epoch 3.

There was also a significant rise in the proportion of patients in whom a troponin test was undertaken in epoch 2 (*p* < 0.001; Figure 2C), with a matching rise in the proportion of patients with a positive troponin test (*p* < 0.001). This was driven by increases from epoch 1 to epoch 2 in the proportion of patients with both dyspnoea and chest pain in whom a troponin test was undertaken (*p* < 0.001 and *p* = 0.001, respectively; Figure 2D). The more dramatic increase in the former coupled with the rapid increase in the number of attendances with dyspnoea together meant that dyspnoea overtook chest pain in epoch 2 as the most common single symptom prompting troponin testing (Figure 2E), before this pattern gradually reverted back to the opposite order in epoch 3 (as in epoch 1). There was no statistically significant change in troponin test positivity rate in patients presenting with chest pain (*p* = 0.79), but there was a trend to an increase in troponin test positivity rate in those presenting with dyspnoea (*p* = 0.07, Figure 2F).

### Change in cardiac procedure rates during COVID-19

**Figure 3:**
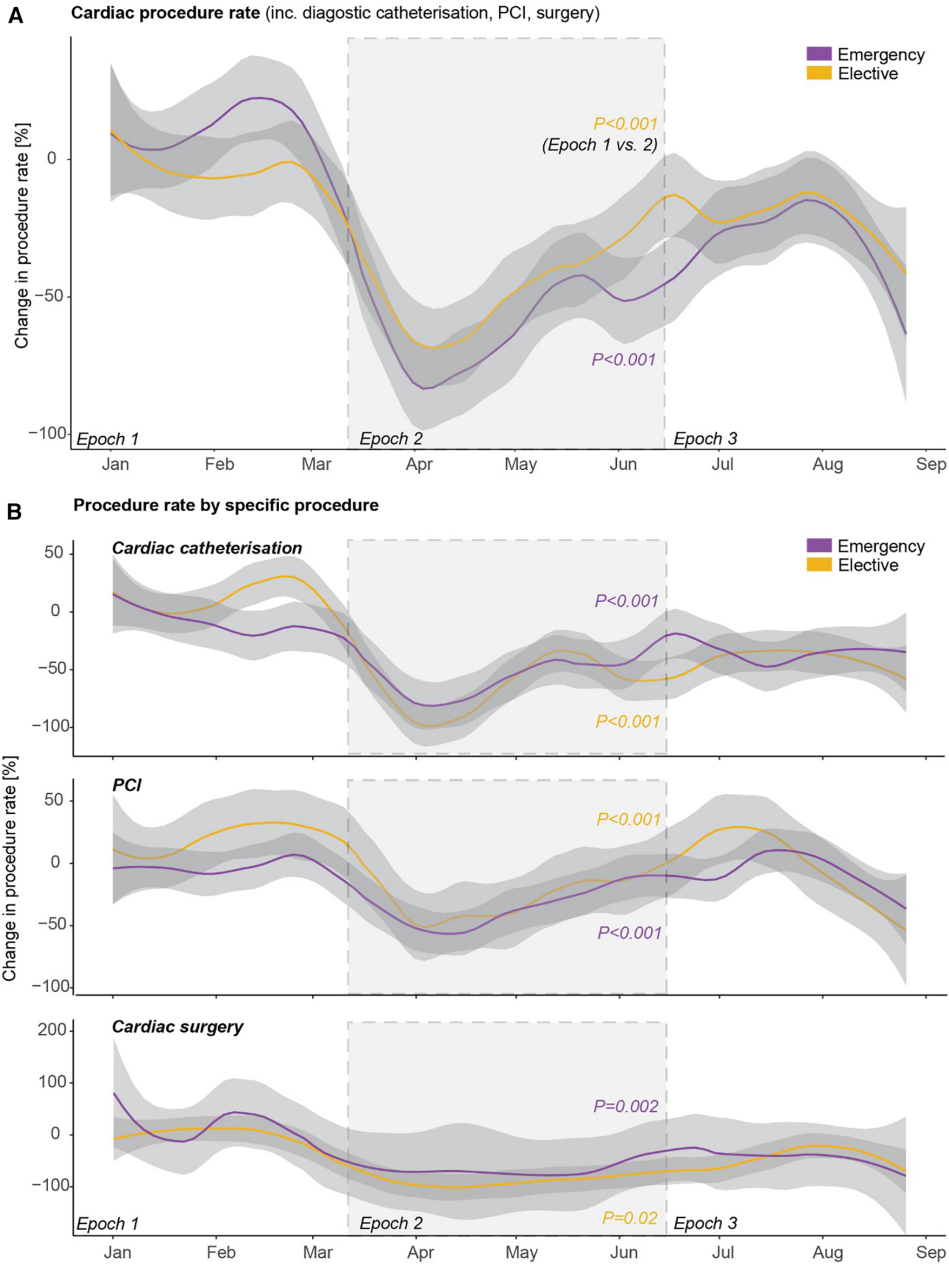
Cardiac procedure rates during the first wave of COVID-19. Trends in rates of (**A**) overall emergency and elective cardiac procedures including diagnostic cardiac catheterisation, percutaneous coronary intervention (PCI), and cardiac surgery, and (**B**) the individual emergency and elective cardiac procedures.

There was a reduction in both elective and emergency cardiac procedures in epoch 2 compared to epoch 1 (both *p* < 0.001; Figure 3A), with partial recovery in epoch 3. This pattern was consistent across elective and emergency cardiac catheterisation, percutaneous coronary intervention, and cardiac surgery (Figure 3B).

### Survival of admitted patients

**Figure 4:**
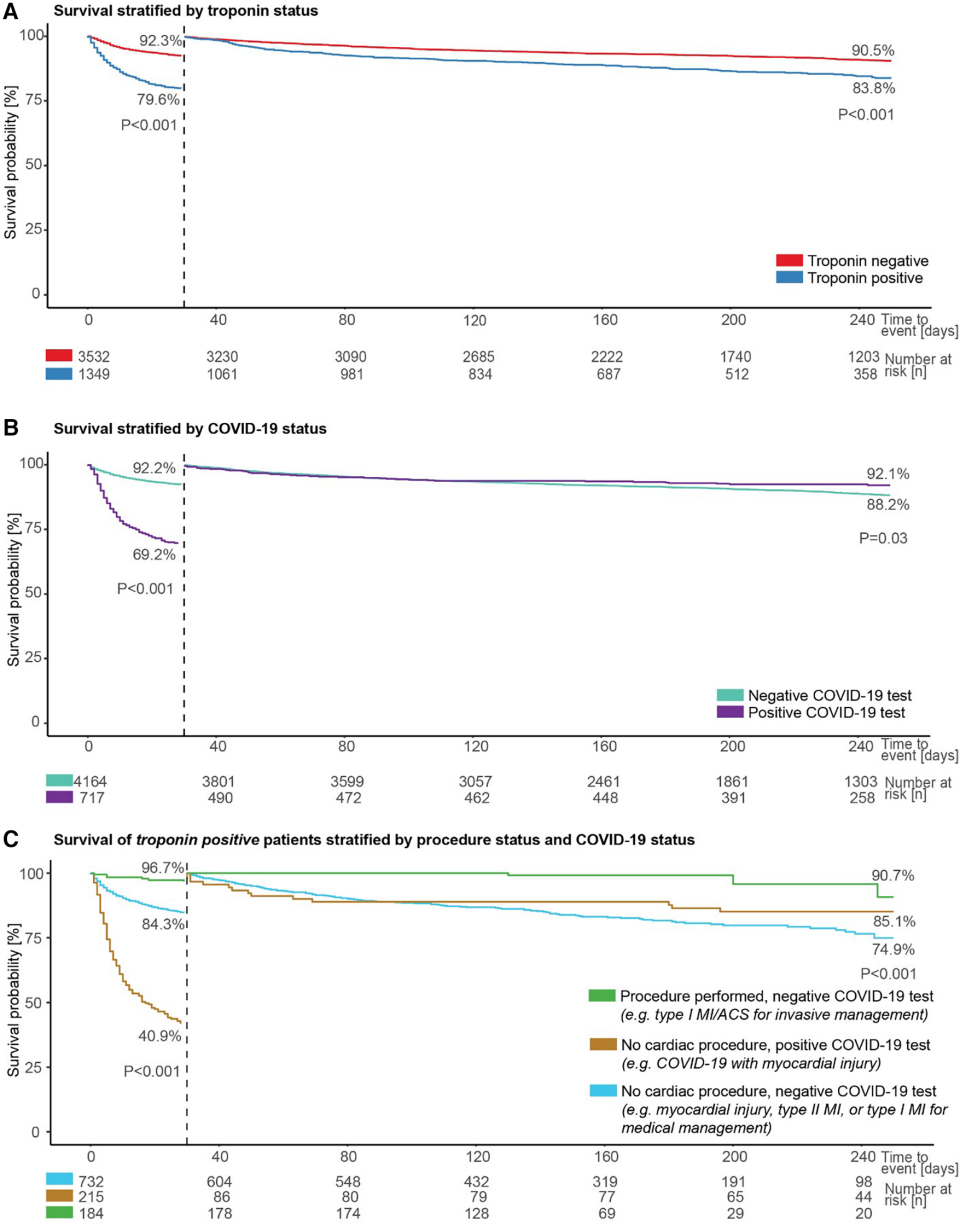
Patient survival by troponin, COVID-19, and cardiac procedure status. Survival of admitted patients (**A**) stratified by troponin status, including landmark analysis from 30 days, (**B**) stratified by COVID-19 status, including landmark analysis from 30 days, and (**C**) stratified by procedure and COVID-19 status in those with a positive troponin test.

Troponin positive status was associated with a higher risk of death in patients admitted after an ED presentation with chest pain or dyspnoea, both in a 30-day landmark analysis and longer-term outcomes when considering death rates up to the end of available follow-up in those alive at 30-days (both *p* < 0.001; Figure 4A).

Similarly, a positive COVID-19 test was associated with a greater risk of death within 30-days in the same cohort of patients compared to individuals with no positive COVID-19 test (*p* < 0.001; Figure 4B). However, when considering death rates starting from 30 days from admission to the end of available follow-up, a positive COVID-19 test was associated with a lower risk of dying (*p* = 0.03; Figure 4B).

Survival was next evaluated for three groups constituted from available data for troponin positive patients in the same cohort, depending on the presence or absence of a positive COVID-19 test and whether or not a cardiac procedure was undertaken. Patients with no positive COVID-19 test and a cardiac procedure are likely, primarily, to represent a group considered to have a type I myocardial infarction or acute coronary syndrome and to be suitable for invasive management, and this group had the best survival (96.7% at 30-days, and 90.7% between 30-days and end of available follow-up; Figure 4C). The worst prognosis in the first 30-days was seen for the group with a positive COVID-19 test and no cardiac procedure; this group was likely to include patients considered to have COVID-19 infection with myocardial injury, and survival was 40.9% at 30-days. The final group, with no positive COVID-19 test and no cardiac procedure, was likely to include individuals considered to have myocardial injury, type II myocardial infarction, or a type I myocardial infarction suitable for medical rather than invasive management, and had the worst survival between 30-days and end of available follow-up (74.9%). The differences between these groups were significant in both analyses (both *p* < 0.001; Figure 4C).

**Figure 5:**
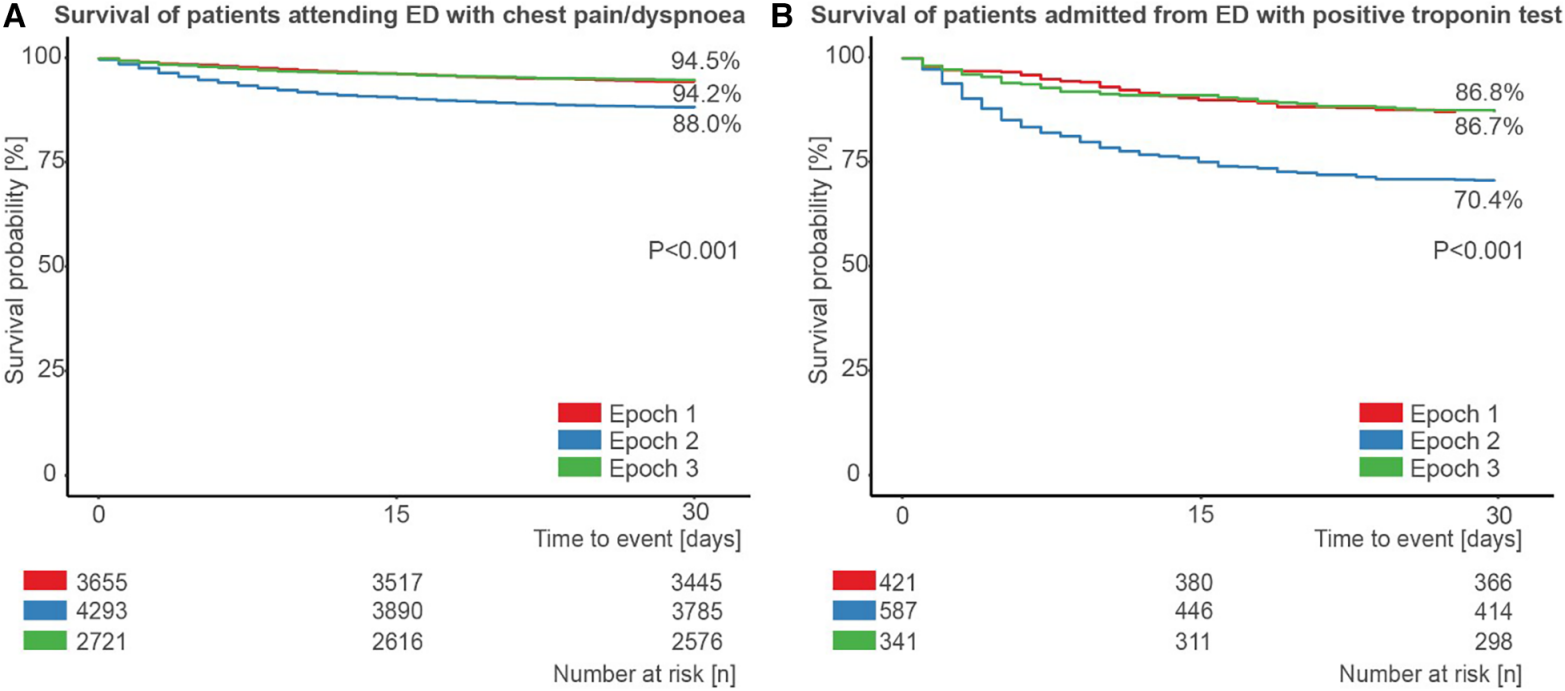
Patient survival during the first wave of COVID-19. Survival of (**A**) patients attending the emergency department (ED) with chest pain or dyspnoea, stratified by epoch, and (**B**) those patients admitted from the ED after attending with chest pain or dyspnoea and with a positive troponin test, stratified by epoch.

Finally, we assessed survival split by time epoch for the 10,670 patients who presented to the ED with chest pain or dyspnoea within the study period (Figure 5A), and for the 1,349 such patients who had a positive troponin test and were admitted (Figure 5B). In both cases, survival was significantly lower in epoch 2 compared to epoch 1 and epoch 3 (all pairwise *p* < 0.001).

## Discussion

We used routine healthcare data from three major UK centres to investigate changes in the presentation, investigation, and management of patients with suspected myocardial infarction or myocardial injury during the first wave of the COVID-19 pandemic in England. Whilst several previous studies have reported that troponin elevation in patients with COVID-19 is associated with adverse outcomes (24–29), we are not aware of any previous large-scale multi-centre studies reporting granular data on decisions regarding the clinical use of troponin testing in this patient population. We demonstrate that the pandemic was associated with significant reductions in the number of patients presenting to EDs, increases in the proportion of patients receiving troponin testing, particularly in patients presenting with a primary complaint of dyspnoea, and reductions in the number of elective and emergency cardiac procedures undertaken. We also show that those patients with myocardial injury or infarction who did not undergo a cardiac procedure experienced worse short- and medium-term survival compared to those who did. Finally, we found significantly higher mortality in patients presenting or admitted during the peak of the first wave of the COVID-19 pandemic in the UK, when compared to the periods immediately before and after this, perhaps reflecting the high rate of adverse outcomes in those patients requiring hospital treatment for COVID-19 infection.

Our data corroborate previous literature documenting a reduction in the number of patients with suspected MI presenting to and admitted to hospital during the COVID-19 pandemic (1, 3–7, 10), and contribute further by demonstrating changes in clinician behaviour with respect to use of troponin testing (increase likelihood of testing in patients with dyspnoea) and invasive cardiac procedures (reduced invasive procedure rate despite higher numbers of patients with positive troponin testing). The drivers of altered patient and clinician behaviour cannot be determined from the available data, but are likely to relate to public health messaging (for patient behaviour) and rapidly evolving medical literature at the time (30–32), including studies which suggested a high risk of and from cardiovascular involvement from COVID-19 in hospitalised patients (17–20, 33). This is likely to be a factor driving greater troponin testing in patients with dyspnoea, but not chest pain.

Our findings highlight differences in the risk profile associated with a positive result from troponin and/or COVID-19 testing. Across the cohort, a positive troponin test was associated with greater risk of mortality at both early (30 day) and medium term (250 day) follow-up, as expected (22, 24–29). A positive COVID-19 test was also associated with a markedly increased risk of mortality at 30-days; however, patients with a positive COVID-19 test who survived the first 30-days had better survival at 250 days than those with a negative COVID-19 test. This most likely reflects the front-loading of risk associated with acute viral infection, in addition to the increased risk of early mortality from COVID-19 in patients with greater comorbidity (34). A similar trend was also present when the survival analysis was restricted to patients with a positive troponin result: in these patients, a positive COVID-19 test was associated with a grave 30-day prognosis, however patients with a positive COVID-19 test who survived the initial 30-days had a paradoxically better prognosis than patients with a negative COVID-19 test who were treated medically rather than undergoing invasive coronary assessment.

The above observations may also be relevant to the finding of worse survival in patients presenting to or admitted from the ED with chest pain or dyspnoea during the peak of the pandemic, compared to the time periods immediately before and after this. One possible explanation is that there was a higher number of patients with COVID-19 (with or without concomitant myocardial injury) during this period, since this group had a higher mortality than patients with myocardial infarction selected for conventional treatment. However, the nature of our study means that we cannot ascribe causality, and the lack of systematic COVID-19 testing throughout the study period precludes more detailed analysis.

Our study had important limitations. The available data was derived from three NHS teaching hospitals, but may not fully reflect the pattern across the entire UK healthcare system. Initial exploration of the dataset demonstrated that the two complaints of chest pain and dyspnoea (annotated using the quoted SNOMED codes) accounted for more than two thirds of all the troponin tests done, with the remainder largely composed of completely different presentations (e.g., palpitation, falls), but it is acknowledged that some patients with suspected myocardial infarction or injury and coded with a different SNOMED complaint code (e.g., “angina”) are not included in the study. Variation in coding practices and the observational nature of the data mean that it is not possible to adjust the observed risks for baseline characteristics and the effects of selection bias, and it is not possible to infer any treatment effect. Furthermore, it was not possible to adjust for small differences in the reference ranges for the different commercial troponin assays used. Finally, as only data on vital status and date of death (rather than cause of death) were available, we were unable to investigate more selected outcomes such as death from cardiovascular causes.

In summary, the first phase of the COVID-19 pandemic in the UK was associated with marked changes in presentation, investigation, and management of patients presenting with suspected myocardial infarction or myocardial injury, with corresponding changes in clinical outcomes. Results from COVID-19 and troponin testing, and clinician decision making with respect to invasive cardiac procedures were all associated with differential mortality risk profiles. These findings may help to inform messaging to patients, the public, and clinicians during future pandemics or other societal crises. More broadly, they also illustrate the potential for research using routinely collected electronic health data to provide detailed insight into changes in clinical practice.

## Conclusion

The first wave of the COVID-19 pandemic was associated with significant changes in the presentation, investigation, management, and mortality of UK patients with suspected myocardial infarction and injury.

## Data Availability

The datasets presented in this article are not readily available because the data presented in this article are accessible via an application to the National Institute for Health Research (NIHR) Health Informatics Collaborative (HIC) Cardiovascular/COVID-19 Theme Scientific Steering Group. Requests to access the datasets should be directed to https://hic.nihr.ac.uk.
